# A simple method of human sperm vitrification

**DOI:** 10.1016/j.mex.2019.09.022

**Published:** 2019-09-18

**Authors:** Dupesh Shah, Rasappan, Shila, Karthik Gunasekaran

**Affiliations:** a VRR Institute of Biomedical Sciences (Affiliated to the University of Madras), India; b The Metromale Clinic, India

**Keywords:** Sperm Vitrification, sperm vitrification, sperm cryopreservation, cryoprotectant, semen freezing, human spermatozoa

## Abstract

Human sperm vitrification is a novel method of sperm freezing which achieves cryopreservation due to ultra-rapid cooling rates that prevent ice-crystal formation. However, sperm vitrification protocols are still largely not standardized for routine clinical use and seldom achieve a post warm sperm survival of 25-35%. The study aim was to validate and optimize a simple method of sperm vitrification that yields a high survival rate of spermatozoa for clinical use. Semen samples from 10 normozoospermic patients were subject to a simple swim-up into pre-warmed gamete handling media. Swim-up specimens were mixed in a 1:1 ratio with 0.5 M sucrose. Swim up specimens were then directly dropped in liquid N2. After a week of storage samples where warmed at 42 degree Celsius and sperm motility and viability was estimated. The mean sperm total motility of the fresh sample after the swim up preparation was 94.3 ± 3.06 %. Upon, vitrification followed by warming the mean percentage of total motile sperm fraction recovered was 74.70 ± 5.60 %. The mean sperm progressive motility of vitrified-warmed spermatozoa was 68 ± 8.47 %. The overall mean percentage of motile sperm recovery was 70.05% of the fresh swim up sample in this study. The overall mean sperm viability as assessed using the HOST vitality test was 77.21 ± 7.52%.

• This study presents a simple protocol on the 'droplet method' of sperm vitrification.
• Sperm cells vitrified using our modified method show a >70% motility and viability rates compared to the routine 25% to 35% of reported survival with the original sperm vitrification/freezing methodologies. This survival is attributed to a crucial change in the warming step.
• This method has the advantage of using no toxic cell permeating cryoprotectant or expensive programmable freezing devices.

## Graphical abstract

**Figure:**
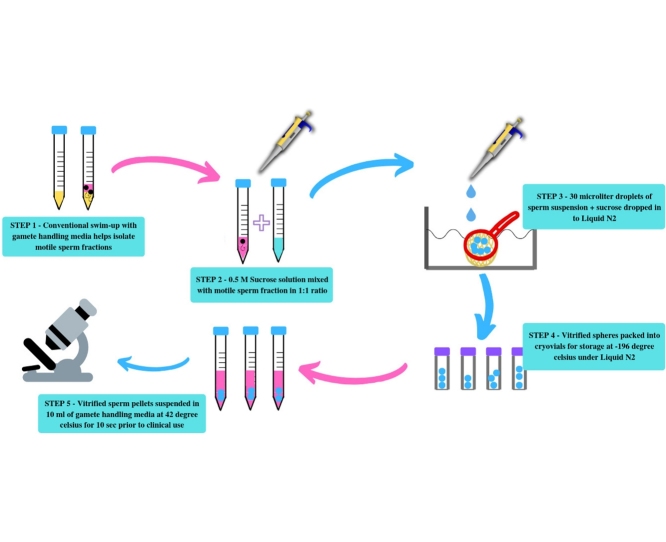

**Specification Table**

**Table:** 

Subject Area:	• *Medicine and Dentistry*
More specific subject area:	*Reproductive Medicine*
Method name:	*Sperm Vitrification*
Name and reference of original method:	*V. Isachenko, E. Isachenko, M. Montag, et al. Clean technique for cryoprotectant-free vitrification of human spermatozoa. Reprod. Biomed. Online. 10 (2005) 350-4* https://doi.org/10.1016/S1472-6483(10)61795-6
Resource availability:	*NA*

## Method details

### Background on the Method

Conventional human sperm cryopreservation is now considered routine in the medical management of male infertility. From preserving extracted testicular spermatozoa for patients with Non-obstructive Azoospermia to routinely preserving spermatozoa for the purpose of sperm banking and/or fertility preservation in case of cancer patients, low temperature storage of human spermatozoa has become a widely used therapeutic intervention in almost all assisted reproductive technology programs [1]. However, cryopreservation have largely remained sub-optimal since the 1950's. Post thaw motility recovery still seldom cross 50% with conventional slow freezing of human sperm [2].

Very little progress has actually been made to modify existing slow freezing protocols or to significantly improve sperm survival rates [3]. Conventional slow freezing of human sperm presents with 2 cardinal problems. The first, is associated with the use of a variety of cell permeating cryoprotectant like dimethyl sulfoxide, glycerol, dimethylacetaldehyde and glycol which cause toxicity due to osmotic stress and also directly affect the spermatozoa's DNA integrity [4]. The second issue, is with the actual rates of cooling and thawing [5]. Suboptimal cooling and warming rates can severely affect the cells survival by a variety of mechanisms, like damage to the cells cytoskeleton, its organelles or plasma membranes or direct damage to the DNA [6,7]. Moreover, rate of cooling and warming also depend on the concentration and/or type of cryoprotectant utilized during slow freezing. A delicate balance between intracellular ice crystal formation and an increasing concentration of dissolved substances in the unfrozen ice fraction of the freezing solution needs to be achieved for optimal cell survival [8]. The human spermatozoa is also particularly sensitive to osmotic shock and low temperatures especially in the range of 0 degree to 20 degree Celsius. This is called 'Cold shock'.

Identifying an optimal cooling rate for the human sperm has been exceedingly difficult despite 50 years of research [9].

Vitrification on the other hand is a method of ultra rapid freezing without the use of permeating cryoprotectant [10].

Interestingly, Luyet [11] in 1937 had first applied the technique of vitrification to frog sperm which was then supported by independent observations of Schafnner in 1941[12]. Subsequent attempts were also made by Hoagland and Pincus [13] in 1942, when they tried to freeze human spermatozoa on bacteriological loops where the authors reported they got over 40% viability.

After this period, the success of vitrification techniques were reported in mouse embryos. Post this, vitrification has been extensively applied and utilized in the cryopreservation of both animal and human eggs and/or embryos [14]. However, a direct extrapolation of these vitrification techniques could not be directly applied to the human spermatozoa due to deleterious effects of high concentration of permeating cryoprotectant used in vitrifications protocols which results in 'osmotic shock' [5]. In recent times newly developed vitrification techniques have been developed which involves direct plunging of spermatozoa into liquid N2 [[15], [16], [17]]. The high rates of cooling (300 - 600 degree Celsius/minute) seemed to have some benefit. Moreover, these methods do not technically require the use of cell permeating cryoprotectant that could result in osmotic shock to the spermatozoa. Prepared swim up or density gradient separated spermatozoa that are preselected are used in order to optimize post thaw sperm quality in terms of progressive motility, percentage of normal morphology and/or DNA integrity [16,17]. The other advantage is that both the freezing and thawing process is complete in minutes. Isachenko and colleagues have shown that their method of sperm vitrification gave similar rates of sperm motility and DNA integrity when compared with conventional slow freezing[17]. The same group have also demonstrated an aseptic technique of sperm virtrification using open pulled straws and also vitrification utilizing cryoloops [18]. Recently a new method of sperm vitrification using a 'cell sleeper device' was shown to work for vitrifying a small number of spermatozoa [19]. Liu's groups have recently shown that direct fumigation of a small number of spermatozoa is comparatively better than slow freezing [20]. Another study on solid surface vitrification showed lower DNA damage of sperm as compared to conventional slow vapour freezing [21]. A recent study compared various semen parameters including hyaluronan binding and DNA fragmentation between sperm vitrification with conventional freezing and concluded that sperm vitrification is not superior to conventional slow freezing of spermatozoa in case of normozoospermic samples [22].

Of more clinical importance though is that vitrified spermatozoa have been utilized in ICSI and healthy twins have been delivered [23]. Another live birth from an IUI procedure was also reported from a patient with oligoasthenozoospermia where the semen was vitrified without the use of cryoprotectant [24]. Sperm vitrification would seem to have a role to play in assisted reproductive technology, but the number of studies done on the subject is still limited. Therefore, more studies on sperm vitrification are required to confirm the validity of the cryopreservation methodology for routine application in ART clinics. Encouraged by such findings we wanted to explore the application of sperm vitrification in our laboratory. The aim of the study was to optimize the vitirification and post warm recovery of spermatozoa in the absence of permeating cryoprotectant for normozoospermic semen samples.

### Method

The study was approved by the institutional ethics committee (VRRIBMS000012937). Semen samples were obtained after both written and informed consent from 10 patients undergoing a male infertility evaluation. Semen samples were collected by masturbation with an ejaculatory abstinence of 3 to 5 days for all patients. Post collection, a routine semen analysis was done as per WHO 2010 guidelines. After semen analysis, the left over sample was used for vitrification. The motile sperm fraction was separated first by a routine swim up method of sperm selection. Briefly, 0.5 ml of standard gamete handling medium supplemented with 10 mg/ml of human serum albumin (Quinn's advantage, REF ART-1023Cooper surgical, Denmark) was overlaid on 1 ml of semen. An incubation time of 45 minutes was allowed for the swim-up procedure. The top 0.4 ml containing the motile sperm fraction was then gently aspirated out leaving the interface and diluted with the same gamete handling medium to achieve a final concentration of 10 × 10^6^/ml. This suspension was then mixed drop by drop with an equal volume (1:1) of freshly prepared 0.5 M sucrose solution with gentle agitation to get a final sperm concentration of 5 × 10^6^/ml.

### Vitrification Procedure

**Fig. 1:**
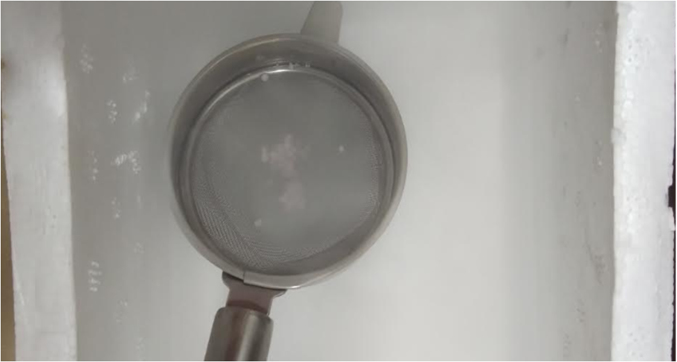
Vitrified spermatozoa form spheres on contact with Liquid N2.

**Fig. 2:**
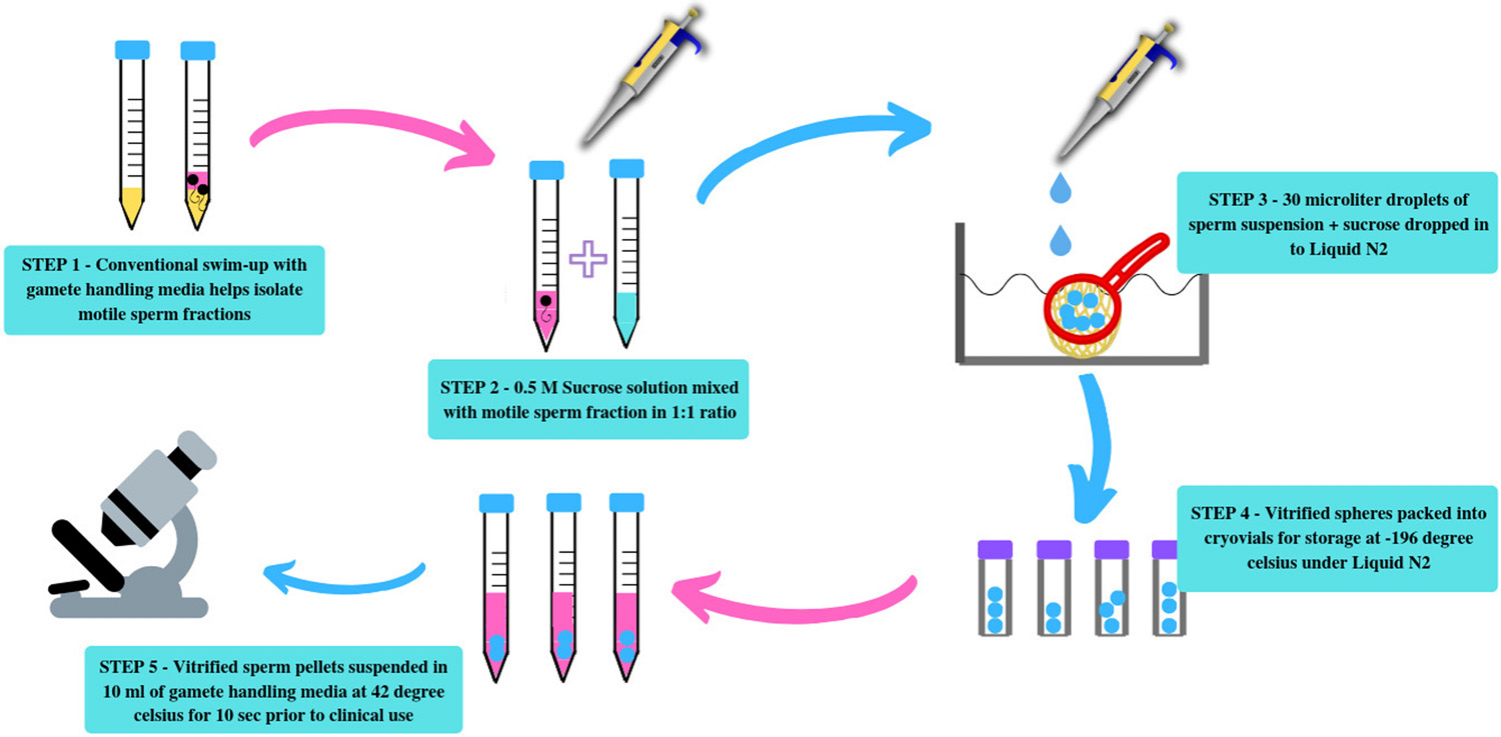
Depicts the various steps of sperm vitrification.

For vitrification we based our the methodology as previously described by Ischachenko et al (2008) with a 2 modifications, the first one was that we used a simple tea strainer instead of the original large metal strainer and cryotube [25]. The second one was the change in the warming temperature which was adjusted to 42 degree Celsius instead of the original 37 degree Celsius as indicated by the study. Briefly aliquots of 30 micro litres of prepared swim up sperm mixed with sucrose solution where directly dropped into liquid N2 which contained a stainless steel tea strainer with the help of a micropipette. The aliquots solidified on contact with the liquid N2 and post solidification, the spheres formed settled down into the strainer, post which the spheres (Fig. 1) where then immediately packaged into cryovials pre-cooled in liquid N2 prior for further storage. For devitrification/warming, the spheres after a period of 1 week of storage were taken out and were submerged in 5 ml of pre-warmed gamete handling media supplemented with human serum albumin at 42 degree Celsius for a total period of 10 seconds (Fig. 2 depicts the protocol). Our choice for using 42 degree Celsius for warming was based on one study by Mansilla et al [26] which found a significant increase in sperm motility and plasma membrane integrity when warming was performed at 42 degree Celsius as compared to 40 degree Celsius or 38 degree Celsius respectively. After warming, an estimation of sperm motility and viability parameters was done using light microscopy at 400 × .

Reagents & Materials Required

### M freshly prepared Sucrose solution

Gamete handling media with HSA 10 mg/ml

Liquid Nitrogen

Thermocol Box

Nunc 2.0 ml cryotubes

Tea Strainer

### Notes on the Protocol

1. Normozoospermic semen samples when directly mixed with 0.5 M sucrose in a 1:1 fashion and then subjected to vitrification also gave over 60% survival and motility on warming. The additional step of sperm preparation (swim-up) that takes over 45 minutes of time can be used more specifically for samples were the motility is low. Direct vitrification of semen also gives good survival in terms of motility and viability as compared to normal sperm freezing. We tried this technique with 5 samples specifically. This technically removes one more major step in the sperm preservation process.

2. When subjecting large volume semen samples (>4 ml), to vitrification, dividing the aliquots into 2 + 2 ml fractions and then subjecting the sample to swim up is recommended, since, a single Nunc cryotube holds 2 ml of volume only. Moreover, once vitrified, a rapid transference of the spheres into the cryovials is required to quickly store the vitrified sperm spheres in liquid N2. With larger volumes, more spheres have be transferred and there is a major risk of spheres undergoing spontaneous thawing in the time that is taken to transfer the spheres from the tea strainer into the cryovial. This step is very crucial to achieve high survival and motility on warming the sample.

3. The 0.5 M sucrose solution should be mixed with either prepared sperm samples or raw semen only drop by drop with gentle agitation. Once an equal volume is mixed, a 30 micolitre volume is taken and held up from liquid N2 in the thermocol box at the height of 20 to 30 cm. The droplets will form spheres on contact with liquid N2, the spheres must be allowed to settle to the bottom of the tea strainer before transferring them to the cryovial for storage.

4. 0.5 M Sucrose is prepared freshly by dissolving 0.5 moles of sucrose with 1 liter of molecular grade double distilled water that is Millipore treated.

### Method Validation & Discussion

The mean sperm total motility of the fresh sample after the swim up preparation was 94.3 ± 3.06 %. Upon, vitrification followed by warming sperm total motility decreased to a mean value of 74.70 ± 5.60 %. This difference was significant (p = 0.0014). Moreover, vitrified and warmed sperm also showed a higher degree of coiled tails and bent necks when compared to the fresh samples (p < 0.05). The mean sperm progressive motility of vitrified-warmed spermatozoa was 68 ± 8.47 %. The overall mean percentage of motile sperm recovery was calculated to be 70.05% of the fresh swim up sample in this study. The overall mean sperm viability as assessed using the HOST test was 77.21 ± 7.52%.

Conventional slow freezing of human spermatozoa still remains largely inefficient. This study explores the fairly new domain of human sperm vitrification. Many years ago attempts were made to vitrify human spermatozoa. Jahnel and Parkes both independently attempted vitrification of human spermatozoa in the 1930's in the absence of permeating cryoprotectant [27,28]. Hoagland and Pincus also reported successful vitrification of both human and rabbit spermatozoa utilizing bacteriological loops to quickly cool and then warm a small volume of sample. These authors reported getting over 40% sperm viability on thaw [13]. In 2002, Isachenko's group [17] reported a simplified method of sperm vitrification without non permeating cryoprotectant use. A 49.5% motile fraction recovery rate was suggested which was significantly higher than the 37.9% recovery rate of conventional slow freezing. The same group in 2005, also suggested different protocols/methods of human sperm vitrification. They compared 4 different techniques of vitrification namely, open pulled straws, droplet method, open straw and the cryoloop methods [16]. No significant differences in terms of post warm sperm motility or viability was observed. Mean percentage of sperm motility recovery ranged from 25-35%. Sataripod study reported a sperm motility rate of 40% after vitrification of raw semen [21]. Only one study by Sanchez reported a 75% sperm motility recovery rate [24]. Depending on the type of protocol and/or technique sperm vitrification recovery rates seem to vary from 25% to 40%.

Attempts at human sperm vitrification was also made with embryo freezing carrier devices like cryotop and cell-sleepers [19]. However, only a small number of spermatozoa could be successfully vitrified on such devices. The straw method of vitrification has been suggested as a method of choice for vitrifying larger volumes of sperm specimens. Studies on vitrified-warmed human sperm have shown similar DNA integrity as compared to conventional slow freezing. Moreover, the safety of sperm vitrification techniques have also been validated with the birth of healthy live offspring from both intra-uterine insemination as well intracytoplasmic sperm injection [23,24]. Thus the data despite being small suggests that the vitrification techniques are safe and can be used in A.R.T clinics in patients treatments.

Our group has successfully demonstrated a simple method of sperm vitrification by modifying the droplet method for both small and large sample volumes. Our study findings demonstrate excellent recovery in terms sperm motility and viability that is >70% for normozoospermic semen specimens. The technique suggests an important modification of the warming step in the original droplet methodology of vitrification. The high recovery rates obtained in our study could be due to the 42 degree Celsius temperature used for warming as compared to 37 degree Celsius in the previous studies of sperm vitrification. Warming places a very critical role in cell survival just as cooling does, since the lethality of cell damage depends on the intermediate zone of temperature (-10 to -60 degree Celsius) and the cells has to traverse this intermediate zone twice during a cryopreservation protocol [29]. While vitrification in the absence of permeating cryoprotectant accounts for both ultra-fast cooling rates and the avoidance of toxic permeating cryoprotectant, the concept of optimizing the warming protocol has largely been ignored or unintentionally missed in previous studies for sperm vitrification.

This study although small in number suggests an optimized yet simple methodology of sperm vitrification in the absence of permeating cryoprotectant based on the original droplet method. This study also confirms the finding of all previous research on human sperm vitrification. This study suggests the simplified droplet method of sperm vitrification with warming at 42 degree Celsius is recommended for routine clinical use. The droplet method is easy, requires no major technical training and can be used in place of conventional slow freezing for normozoospermic semen samples. Post thaw sperm motility and viability is in general excellent. The study is not without its limitations though, the study size is small, and the sperms DNA integrity was not assessed. Much larger studies on sperm vitrification with more data on the sperms DNA integrity and separate protocols for suboptimal semen samples are required for wide scale use of the technique. We are currently evaluating the feasibility of applying the technique to oligozoospermic and asthenozoospermic samples.
